# Alcohol consumption may be associated with postoperative delirium in the elderly: the PNDABLE study

**DOI:** 10.1186/s12871-023-02178-x

**Published:** 2023-06-23

**Authors:** Xiaoyue Wu, Ning Zhang, Bin Zhou, Siyu Liu, Fei Wang, Jiahan Wang, Xinhui Tang, Xu Lin, Bin Wang, Yanlin Bi

**Affiliations:** 1 Department of Anesthesiology, Qingdao Hospital, University of Health and Rehabilitation Sciences (Qingdao Municipal Hospital), 5, Dong-Hai Middle Road, Shi-Nan District, 266000 Qingdao, China; 2 Department of Anesthesiology, Qingdao Eighth People’s Hospital, Qingdao, China

**Keywords:** Elderly, Alcohol consumption, Alzheimer-related biomarkers, Cerebrospinal fluid

## Abstract

**Objectives:**

This study aimed to reveal the relationship between alcohol consumption and Postoperative delirium (POD) in the elderly.

**Methods:**

We selected 252 patients from the Perioperative Neurocognitive Disorder And Biomarker Lifestyle (PNDABLE ) study. Patients in the PNDABLE database have been measured for Alzheimer-related biomarkers in CSF (Aβ_40_, Aβ_42_, P-tau, and tau protein). Mini-Mental State Examination (MMSE) was used to assess the preoperative mental status of patients. POD was diagnosed using the Confusion Assessment Method (CAM) and assessed for severity using the Memorial Delirium Assessment Scale (MDAS). Logistic regression analysis was utilized to explore the association of alcohol consumption with POD. Linear regression analysis was used to study the relationship between alcohol consumption and CSF biomarkers. Mediation analyses with 10,000 bootstrapped iterations were used to explore the mediation effects. Finally, we constructed the receiver operating characteristic (ROC) curve and the nomogram model to evaluate the efficacy of alcohol consumption and CSF biomarkers in predicting POD.

**Result:**

The incidence of POD was 17.5%. Logistic regression showed that alcohol consumption (OR = 1.016, 95%CI 1.009–1.024, *P* < 0.001) is a risk factor for POD. What’s more, Aβ_42_ is a protective factor for POD (OR = 0.993, 95%CI 0.989–0.997, *P* < 0.05), and P-Tau was a risk factor for POD (OR = 1.093, 95%CI 1.022–1.168, *P* < 0.05). Linear regression analysis revealed that alcohol consumption was negatively associated with CSF Aβ_42_ (β = -0.638, *P <* 0.001) in POD patients. Mediation analyses showed that alcohol consumption is likely to partially mediate POD through Aβ42 (proportion:14.21%). ROC curve showed that alcohol consumption (AUC = 0.904; *P* < 0.001) exhibited a relatively better discriminatory ability in POD prediction compared to Aβ_42_ (AUC = 0.798; *P* < 0.001). The calibration curve indicated a good nomogram prediction (*P* = 0.797).

**Conclusion:**

Alcohol consumption is a risk factor for POD (particularly for those with > 24 g a day on average) in the elderly, and contributes to POD through the mediation of Aβ_42_.

## Introduction

Postoperative delirium (POD) can be defined as an acute cerebral dysfunction or failure and fluctuating consciousness, accompanied by obvious impairment of concentration and cognitive function [1, 2]. It often occurs in the first week after surgery (or before discharge) with a higher incidence 1–3 days postoperatively [3]. POD may lead to higher health resource costs and mortality and longer hospitalization [4]. POD remains a common postoperative clinical complication in the elderly. Previous studies have shown that the incidence of POD is about 17.6% [5] and its pathological mechanism has yet to be fully understood.

With the rapid growth of the Chinese economy, alcohol consumption in China is greatly rising [6]. According to epidemiological findings, alcohol consumption may increase the risk of cognitive impairment in the elderly [7]. Studies have also shown that alcohol consumption is considered to be an independent risk factor for cognitive impairment [8]. Since POD is often accompanied by cognitive impairment [4], a possible correlation between alcohol consumption and POD can be inferred. Considering the current drinking status, actively exploring the relationship between alcohol consumption and POD is imperative to prevent the occurrence of POD in China.

Previous studies have shown that CSF Aβ_40_, Aβ_42_, T-tau and P-tau are associated with neurological abnormalities [9–11]. These markers are usually associated with the pathogenesis of POD [10, 12, 13]. Aβ interfering with synaptic function by binding to different neuronal or non-neuronal plasma membrane components is the basis for clinical manifestations of cognitive decline [14]. After phosphorylation of the tau protein, it loses the function of stabilizing the microtubule cytoskeleton, leading to neurodegenerative diseases [15]. It was also found that patients with POD had a lower Aβ_42_/P-tau ratio and a lower CSF Aβ_42_ level [12, 16, 17]. Thus, the above conclusions can be intertwined to conclude that CSF biomarkers and cognitive dysfunction are related.

There is little research on the relationship between alcohol consumption and POD in the elderly and their related mechanisms. This study was analyzed in the following three aspects to reveal their relationship. First, is alcohol consumption an independent influence on POD? Second, is there a correlation between CSF biomarkers and alcohol consumption? Second, is there a correlation between CSF biomarkers and alcohol consumption? Third, if a correlation does exist, whether alcohol consumption will lead to POD through CSF biomarkers. If the above conjecture were confirmed, the study would hopefully provide a preemptive strategy to decrease the incidence of POD in the elderly and reduce the burden on families and society.

## Materials and methods

### PNDABLE database

Volunteers were recruited from the PNDABLE study, which is an ongoing large-scale cohort study launched in 2018, and volunteers included in the database were between 40 and 90 years of age, concentrating on the risk factors and biomarkers of perioperative neurocognitive disorder (PND) in the Han population of northern China. The purpose of PNDABLE is to determine the genetic and environmental factors of PND biomarkers and the lifestyle factors that might change the risk of PND in the non-demented northern Chinese Han population so that the basis for disease prevention and early diagnosis can be formed. All participants were provided informed consent, and they could stop participating anytime for any reason. Their CSF and blood samples could be used for research in the future.

### Participants

At Qingdao Municipal Hospital, we selected patients who underwent knee/hip replacement surgery under Combined spinal-epidural anesthesia between June 2020 and June 2021. The inclusion criteria of this study include (1) aged between 65 and 90; (2) Drinking frequency ≥ 1 time per week and a history of drinking ≥ 1 year [18]. (3) American Society of Anesthesiologists (ASA)I~II; (4) preoperative cognitive status was intact with no language communication barrier. The exclusion criteria included: (1) central nervous system infection, head trauma, multiple sclerosis, neurodegenerative diseases (such as epilepsy, Parkinson’s disease), or other notable neurological diseases; (2) severe visual and hearing impairment (3) non-drinkers, abstainers, or those who used to drink regularly but have not consumed alcohol in the past year; (4) preoperative Mini-Mental State Examination scale (MMSE) ≤ 23 points (5) drug abuse or psychotropic substance abuse, long-term use of steroids and hormonal drugs, (6) family history of genetic disorders (e.g., early-onset familial AD, hereditary ataxia, hereditary spastic paraplegia, etc.).

### Neuropsychological testing

All participants accepted careful clinical and neuropsychological assessments and MMSE the day before the scheduled operation. Patients were followed up on postoperative days 1–7 days or before being discharged from the hospital at 10 a.m. and 2 p.m. twice daily. At the same time, the presence or absence of POD was recorded. The presence of POD was defined according to Confusion Assessment Scale (CAM), those with POD were classified as the POD group, and those with POD negative were classified as the non-POD group(NPOD). The severity of POD was defined according to the Memorial Delirium Assessment Scale (MDAS) [19, 20]. All of the above assessments were performed by an anesthesiologist and a neurologist who did not know the patient’s perioperative management(The anesthetist and neurologist who visit preoperatively and postoperatively are different). The CAM and MDAS apply to patients with good credibility and utility [21, 22].

### Anesthesia and surgery

All participants performed elective surgery under combined spinal and epidural anesthesia. The participants did not receive preoperative medications and were instructed not to drink for 6 h or eat for 8 h before surgery. After entering the operating room, we routinely monitored ECG, SpO2, and NBP and opened vein access. The anesthesia position was lateral decubitus, with the space between the spinous processes of lumbar 3–4 (L3-L4) as the puncture site. After a successful puncture, 2ml of cerebrospinal fluid was extracted from the subarachnoid space, followed by 2-2.5ml Ropivacaine (0.66%) injection for about 30 s. The patient’s anesthesia level was controlled below the thoracic 8 (T8). The patient’s oxygen saturation, pulse, blood pressure, electrolytes, etc., were checked regularly (every 3 min) during anesthesia and surgery. After the operation, the patient was sent to the anesthesia recovery room for observation for thirty minutes and returned to the ward if there was no abnormality. Postoperatively, the Numerical Rating Scale (NRS) was used to assess the pain. Patient-controlled intravenous analgesia (PCIA) was used in postoperative pain management. (Butorphanol tartrate injection 10 mg + Toranisetron hydrochloride injection 5 mg + 0.9% sodium chloride solution 89ml maintained NRS < 3 points).

### CSF core biomarkers measurements and collection

2ml CSF was collected in a polypropylene centrifugal tube, then centrifuged at 2000 × g for 10 min at room temperature [23, 24] as well as separated and stored in an enzyme-free EP (Eppendorf) tube (oxygen bottle, PCR-02-C) at -80 °C for further use in the following steps of this study. These samples were subjected to at most two freeze-thaw cycles.

ELISA was used to detect the level of Aβ_40_, Aβ_42_, T-tau and P-tau, which were detected from 2ml CSF, using Aβ_40_ (BioVendor, Ghent, Belgium Lot: No. 292–6230), Aβ_42_ (BioVendor, Ghent, Belgium Lot: No. 296-64401), P-tau (BioVendor, Ghent, Belgium Lot: QY-PF9092), and tau (BioVendor, Ghent, Belgium Lot: No.EK-H12242) assay kit under the manufacturer’s instructions. Finally, using an enzyme marker (EnSpire, PerkinElmer, Waltham, MA, USA) [23, 24] to measure each hole’s optical density value (OD value) at the wavelength of 450 m. The same laboratory personnel measured all samples, and the group assignment blinded them.

### Classification of alcohol intake

We traced the patient’s drinking history and calculated the average daily alcohol intake according to the formula: the amount of alcohol consumed (g) = amount of alcohol consumed (ml) × alcoholic concentration (%) × 0.8 (The density of alcohol is known to be 0.8 g/cm³). The patients’ drinking history was investigated and classified according to the following criteria: [25].

1. Average daily intake of alcohol < 12 g (mild).
2. Average daily intake of alcohol 12–23 g (moderate).
3. Average daily intake of alcohol > 24 g (heavy).

### Sample size estimation

The preliminary test in this study explored that four covariates(alcohol consumption, Aβ_40_, Aβ_42_, and P-tau)were included in the Logistic regression. According to the previous studies, the POD incidence was 17.6%^5^, and the loss of follow-up rate was assumed to be 20%. Thus, according to the logistic regression events per variable (EPV) sample size calculation method [26], EPV set to 10, the required sample size was 284 cases (4 × 10 ÷ 0.176 ÷ 0.8 = 284).

### Statistical analysis

The Kolmogorov-Smirnov (KS) test was used to determine the normality of the samples. Data that conformed to the normal distribution were expressed as mean ± standard deviation (SD), and data that did not conform to the normal distribution were expressed as the median and 25–75 percentile (M,(Q25, Q75)) or number (%). The two independent samples t-test was used to test whether there was a significant difference in the levels of CSF biomarkers and alcohol consumption between the POD and NPOD groups. The difference was considered statistically significant at *P* < 0.05.

Binary logistic regression was used to discuss whether alcohol consumption independently influenced POD. Moreover, to investigate the range of alcohol intake that predisposes to POD, the average daily alcohol intake of participants was also categorized according to the above-average daily alcohol consumption and included sequentially in logistic regression for the study. Linear regression models examined the relationship between CSF biomarkers and alcohol consumption. The covariates in the binary logistic regression include average daily alcohol intake, Aβ_40_, Aβ_42_, and P-Tau protein, because they were significantly correlated with POD in the univariate analysis (*P* < 0.05). Subsequently, to improve the accuracy of the results, we further corrected for the effect of confounding factors, including age, gender, years of education, cigarette use (yes or no), hypertension (yes or no), Coronary heart disease(yes or no), diabetes(yes or no) and MMSE, which showed that the results were barely changed in this analysis (OR = 1.016, 95%CI 1.009–1.024, *P <* 0.001); A two-way ANOVA was used to investigate the effects of gender and alcohol consumption on POD.

Moreover, linear regression models covering three equations were performed to examine whether the CSF biomarkers mediated the association between Alcohol consumption and POD. Mediation effects were established if the following criteria were simultaneously reached:

1. Changes in alcohol consumption had a significant effect on CSF biomarkers.
2. Changes in CSF biomarkers were responsible for changes in POD.
3. Changes in Alcohol consumption were significantly or not significantly related to POD.
4. The association between Alcohol consumption and POD was attenuated when the CSF biomarkers were added to the regression model.

Furthermore, the attenuation or indirect effect was estimated, with the significance determined using 10,000 bootstrapped iterations. The indirect effect (IE) was *P* < 0.05, considered significant.

The predictive value of Alcohol consumption and the CSF biomarkers was described with a receiver-operating characteristics (ROC) curve, and the area under the curve (AUC) reported the discriminatory ability. Nomogram will be used to visualize the predicted results, and the calibration curve will be used to verify the predicted model.

The data were analyzed with R4.4.1 (R Foundation for Statistical Computing, Vienna, Austria), GraphPad Prism version 8.0 (GraphPad Software, Inc, LaJolla, CA, USA) and Stata MP16.0 (Solvusoft Corporation, Inc, Chicago, Illinois, USA).

## Results

### Participant characteristics

**Fig. 1:**
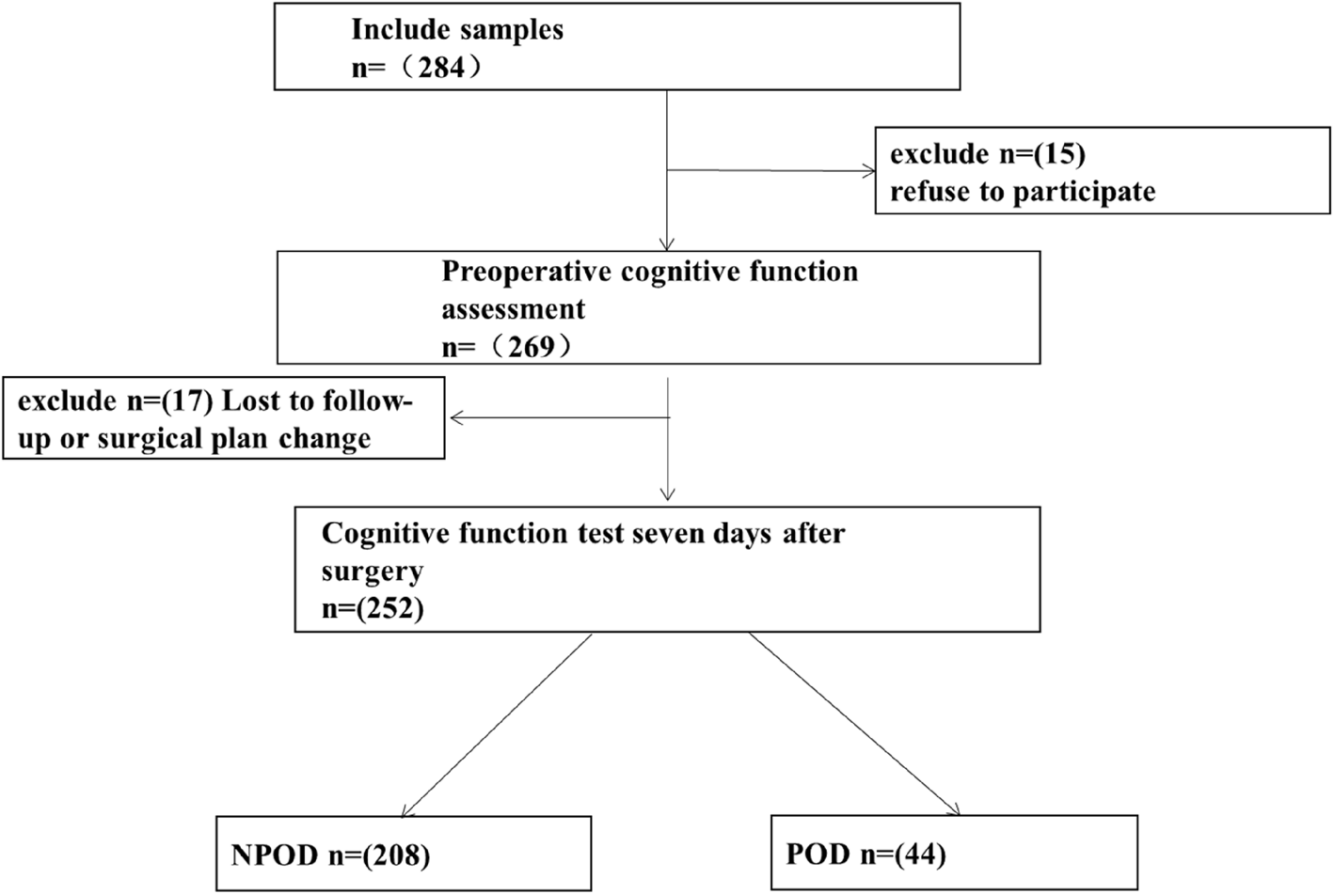
The flow diagram showed the selection of eligible patients and the enrollment process

We eventually included 252 patients for statistical analysis. (see Fig. 1, flow diagram).

Among the 252 patients included in the study, 44 subjects experienced POD during the 1–7 day postoperative follow-up (positive rate of POD: 17.5%). The average age of participants was 69.38 (SD = 4.066). Specific information for all participants was presented in Table 1.

**Table 1:** Characteristics of participants

	POD	Non-POD	t-values/z/χ2		*P*-values
Gender (male/female)	0/44	18/190	2.900		0.089
Age (year),(mean ± SD)	70.20 ± 5.201	69.21 ± 3.776	-1.201		0.235
BMI(mean ± SD,Kg/m^2^)	25.02 ± 3.099	25.45 ± 3.10	0.911		0.363
Education (M,(Q_25_,Q_75_))	9(6,12)	9(9,12)	2.197		0.029
ASA grade, (N(%))			-0.150		0.881
I	10(22.7%)	57(27.4%)			
II	34(77.3%)	151(72.6%)			
NRS, (M,(Q_25_,Q_75_))	3(2,3)	2(2,3)	-1.050		0.295
MMSE, (M,(Q_25_,Q_75_))	27.5(26,29)	29(27,30)	2.838		0.005
MDAS, (M,(Q_25_,Q_75_))	13(11,15.75)	3(0,5)	-25.169		0.000
Cigarette use, yes (%) No(%)^a^	28(63.6%) 16(36.4%)	129(62%) 79(38%)	0.040		0.841
Hypertension, yes (%) No(%)^a^	19(43.2%) 25(56.8)	61(29.3%) 147(70.7%)	3.217		0.073
Diabetes mellitus, yes (%) No(%)^a^	3(6.8%) 41(93.2%)	19(9.1%) 189(90.9%)	0.040		0.841
CHD, yes (%) No(%)^a^	3(6.8%) 41(93.2%)	27(13%) 181(87%)	1.315		0.251
type of surgery (Knee arthroplasty/Hip arthroplasty)	33/11	155/53		1.000	0.943
Surgery time(M,(Q_25_,Q_75_))	1.50(1.03,1.80)	1.40(1.10,1.70)		0.758	0.082
Anesthesia time(M,(Q_25_,Q_75_))	1.95(1.50,2.30)	1.90(1.50,2.18)		0.371	0.064
mean daily alcohol intake(N,(%))				-8.321	0.000
< 12 g		74(35.6%)			
12 ~ 23 g	3(6.8%)	61(29.3%)			
> 24 g	41(93.1%)	73(35.1%)			

Categorical variables are reported as numbers and percentages; continuous variables are reported as means ± SD, whereas non-normal data are expressed as the M(Q_25_, Q_75_);

*Abbreviations*: *POD *Postoperative delirium, *NPOD *No postoperative delirium, *MDAS *Memorial Delirium Assessment Scale, *NRS *Numerical Rating Scale, *CHD *Coronary heart disease, *kg *kilogram, *M *Median, *SD *Standard deviation, *Q *Quartile, *N *Number, *g *gram;

^a^control group

**Table 2:** Logistic regression analysis

		Unadjusted			Adjusted	
	OR	95%CI	*P* value	OR	95%CI	*P* value
**A**						
mean daily alcohol intake	1.016	1.009–1.023	*P* < 0.001	1.015	1.009–1.023	*P* < 0.001
Aβ_40_(pg/ml)	1.000	0.999-1.00	*P* < 0.05	1.000	0.999-1.000	*P* < 0.05
Aβ_42_(pg/ml)	0.992	0.988–0.997	*P* < 0.05	0.992	0.987–0.997	*P* < 0.05
P-tau(pg/ml)	1.076	1.011–1.144	*P* < 0.05	1.073	1.008–1.142	*P* < 0.05
T-tau(pg/ml)	1.005	0.997–1.013	*P* > 0.05	-	-	*-*
**B**						
< 12g	-					
12-23g	0.746	0.482-1.155	*P*> 0.05	0.000	0.000-0.000	*P*> 0.05
>24g	1.014	1.008-1.021	*P*< 0.05	1.011	1.003-1.019	*P*< 0.05

Binary logistic regression was used to discuss whether the amount of alcohol intake was an independent influence on POD. Unadjusted: covariates including mean daily alcohol intake, Aβ_40_, Aβ_42_, P-tau, T-tau；Adjusted: through adding more covariates, including age, gender, years of education, BMI, which showed that the results were barely changed in this analysis

The average daily alcohol intake was transformed into a rank variable according to the above classification ; unadjusted ：Relationship between alcohol classification and POD of the same grade; Adjusted: through adding more covariates, including age, gender, years of education, cigarette use (yes or no), hypertension (yes or no), Coronary heart disease(yes or no), diabetes(yes or no) and MMSE, which showed that the results were barely changed in this analysis

Patients with a rank variable of 1 were not included in this regression analysis because POD did not occur in patients with an average daily alcohol intake of < 12 g.

*Abbreviations*: *OR *Odds ratio, *CI *Confidence interval;

**Table 3:** The simple effect analysis

	I	J	I-J	*P* values	95%CI	
Female	< 12 g	12–23	152.701	0.038	8.822	296.580
A						
	12–23 g	< 12	-152.701	0.038	-296.580	-8.822
male	< 12 g	12–23	-19.629	0.858	-86.026	46.768
		> 24	73.248	0.007	16.228	130.268
	12–23 g	< 12	19.629	0.858	-46.768	86.026
		> 24	92.877	0.001	33.956	151.799
	> 24 g	< 12	-73.248	0.007	-130.268	-16.228
		12–23	-92.877	0.001	-151.799	-33.956
B						
< 12 g	Female	male	113.797	0.022	16.554	211.039
	male	Female	-113.797	0.022	-211.039	-16.554
12-23 g	Female	male	-58.534	0.334	-177.716	60.648
	male	Female	58.534	0.334	-60.648	177.716

The result of simple effect: Fixed gender ; 3B: I-J, the difference in Aβ_42_ between the same gender and different average daily alcohol intake. In this study, women drank within 23g of alcohol, so alcohol levels >24g are not listed in the table

The result of simple effect: Fixed alcohol consumption; 3A: I-J, the difference in Aβ_42_ between genders with the same average daily alcohol intake. In this study, those who drink > 24g are men, so they are not listed in the table

### Comparison between POD and NPOD groups

**Fig. 2:**
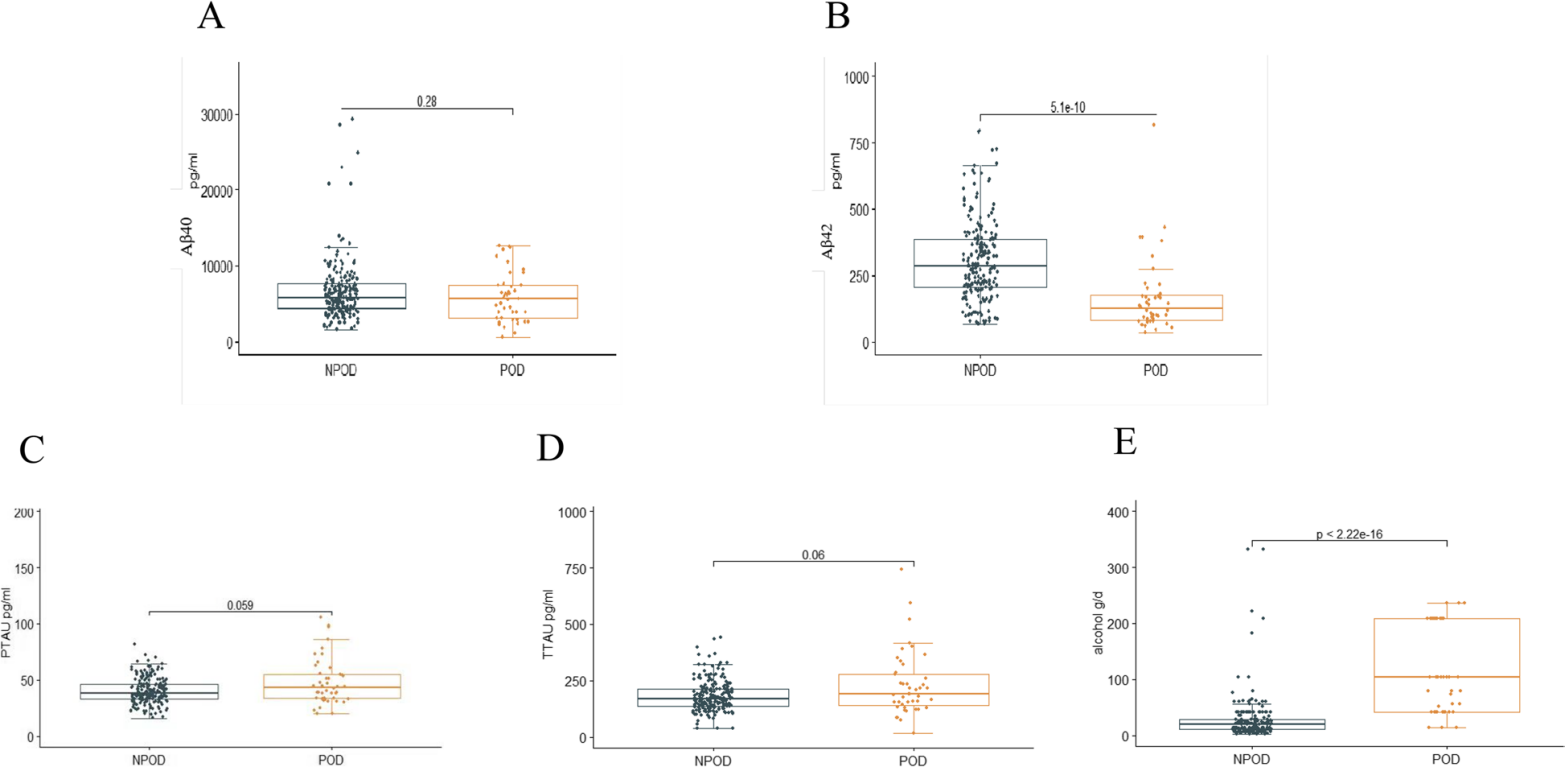
Expression of CSF biomarkers and alcohol consumption of POD patients and non-POD controls. The scatter plots showed the expression levels of Aβ_40 _(**A**), Aβ_42 _(**B**), P-tau (**C**), T-tau (**D**) and alcohol consumption (**E**). The colors of scatter maps are grouped according to different diagnostic groups. The *P* value was determined by the two independent samples t-test. The level of CSF Aβ_42_ and alcohol consumption in patients with POD (POD group) were significantly different than those in patients without POD (NPOD group) (*P* < 0.001)

Participants were divided into two groups according to the occurrence of POD (POD and NPOD), and two independent samples t-test was performed to compare the levels of CSF biomarkers and alcohol consumption. The results suggested a significant difference in the levels of CSF Aβ_42_ protein and alcohol consumption between the POD and NPOD groups (*P* < 0.001). The NPOD group had higher Aβ_42_ levels, while the POD group had more alcohol consumption. However, there was no significant difference in Aβ_40_, T-tau, and P-tau. For the results of the study, please refer to Fig. 2.

### Results of binary logistic regression and sensitivity analysis

Binary logistic regression showed that alcohol consumption (OR = 1.016, 95%CI 1.009–1.024, *P* < 0.001) was a risk factor for POD, and the positive rate of POD would increase with the daily intake (please refer to Table 2A). Subsequently, alcohol intake was further classified according to the *Classification of alcohol intake* and sequentially included in logistic regression for the study. Results showed that an average daily alcohol intake > 24 g (heavy) was significantly associated with the incidence of POD ( OR = 1.014, 95%CI 1.008–1.021, *P* < 0.05 ) (please refer to Table 2B). What’s more, Aβ_42_ was a protective factor for POD (OR = 0.993, 95%CI 0.989–0.997, *P* < 0.05); P-Tau was a risk factor for POD (OR = 1.093, 95%CI 1.022–1.168, *P* < 0.05).

To improve the reliability of the results, we performed a sensitivity analysis: adding more confounding factors, including age, gender, years of education, cigarette use (yes or no), hypertension (yes or no), coronary heart disease(yes or no), diabetes(yes or no) and MMSE. The conclusion was that the results were barely changed in this analysis (OR = 1.016, 95%CI 1.009–1.024, *P <* 0.001).

### The relationship between alcohol consumption and CSF biomarkers

**Fig. 3:**
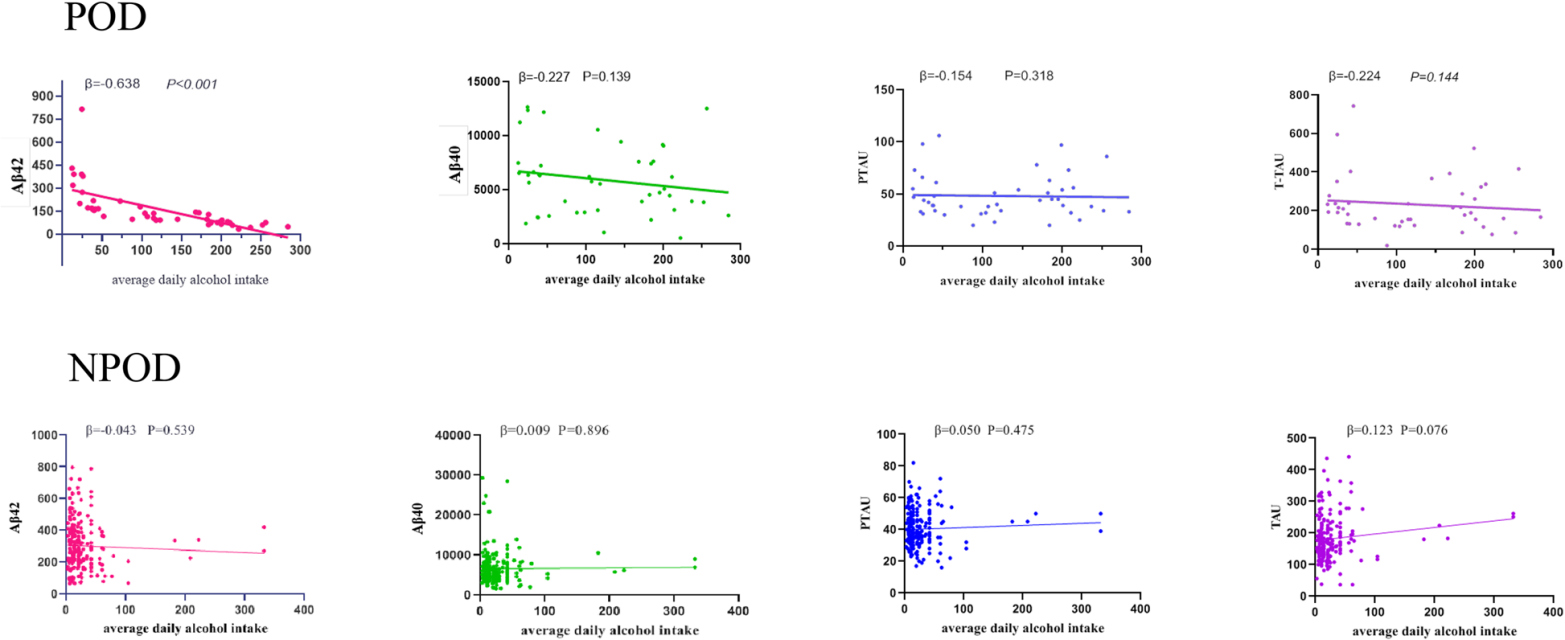
Associations of average daily alcohol intake and CSF core biomarkers. Scatter plots represent the associations of average daily alcohol intake with CSF core biomarkers: Aβ_40_, Aβ_42_, T-tau, and P-tau in the different groups (whole cohort, POD, NPOD). The normalized regression coefficients (β) and P values computed by multiple linear regression after adjustment for age, years of education, BMI, and MMSE were shown

Linear regression analysis showed a significantly negative association between alcohol consumption and CSF Aβ_42_ (β = -0.638, *P <* 0.001) in POD patients, while no such relationship was found in NPOD patients(*P >* 0.05). At the same time, the same relationship was not found for the other three CSF biomarkers(Aβ_40_, P-tau, T-tau *P >* 0.05), regardless of the POD or NPOD group. Relevant graphs of the linear regression results are shown in Fig. 3.

### The mediation analyses

**Fig. 4:**
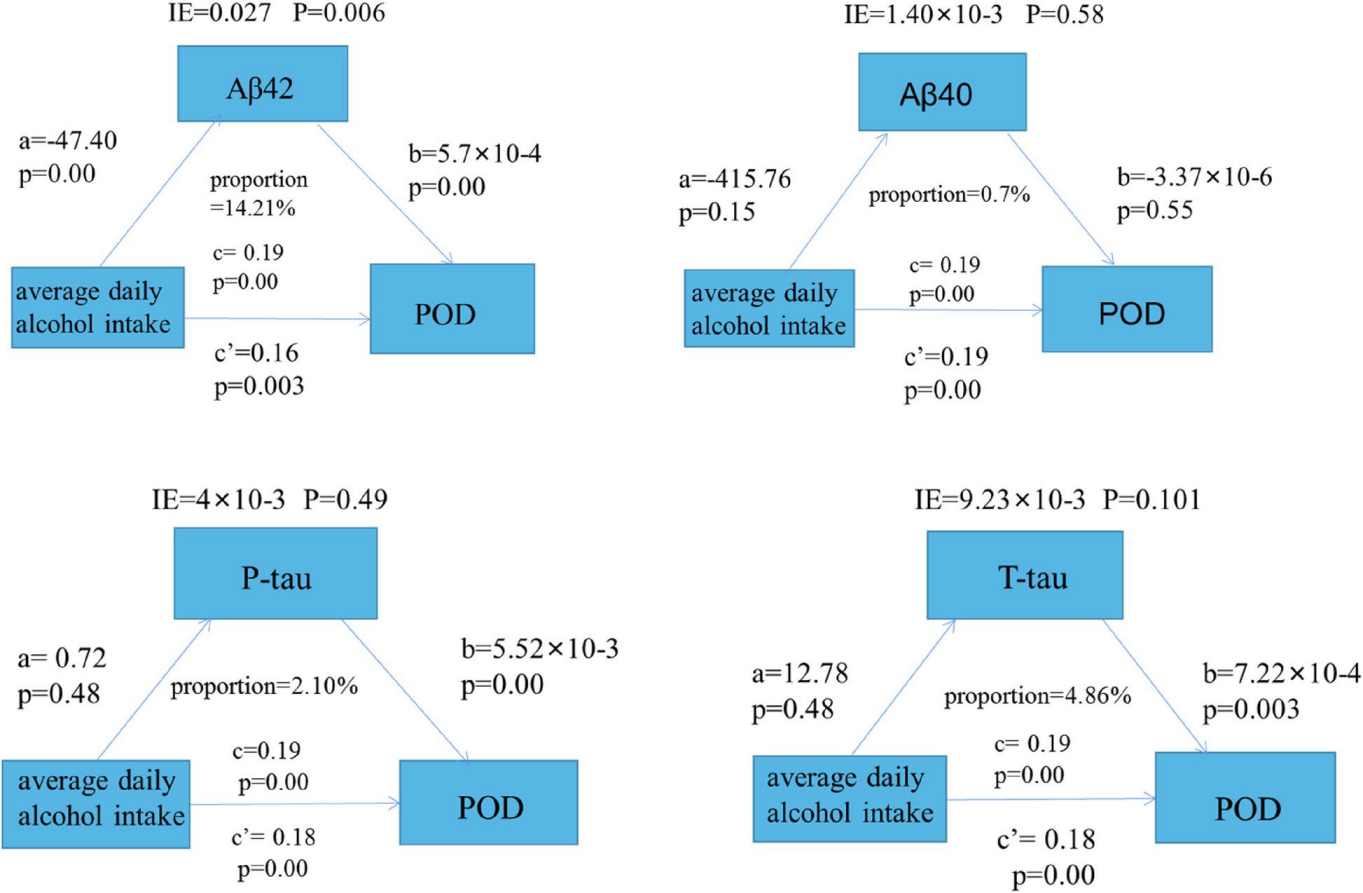
Mediation analyses with 10,000 bootstrapped iterations were used to examine the mediation effects of Aβ, and T-Tau on POD

Mediation analyses showed that alcohol consumption was likely to cause POD through Aβ_42_ (proportion:14.21%, *P* < 0.05). Relevant results are shown in Fig. 4.

### Two-factor ANOVA

A two-factor ANOVA was used to explore the effects of gender and alcohol consumption on Aβ_42_. The results showed a significant interaction between gender and alcohol consumption (*P* < 0.05), and a simple effect analysis performed showed that the level of Aβ_42_ in CSF decreased with increasing alcohol intake when “gender” was fixed (please refer to Table 3A). When the amount of alcohol consumed was fixed, there was no significant effect of gender on the level of CSF Aβ_42_. However, when light alcohol consumption (average daily intake of alcohol < 12 g) was present, women had higher Aβ_42_ levels than men (please refer to Table 3B).

### Predictive model

**Fig. 5:**
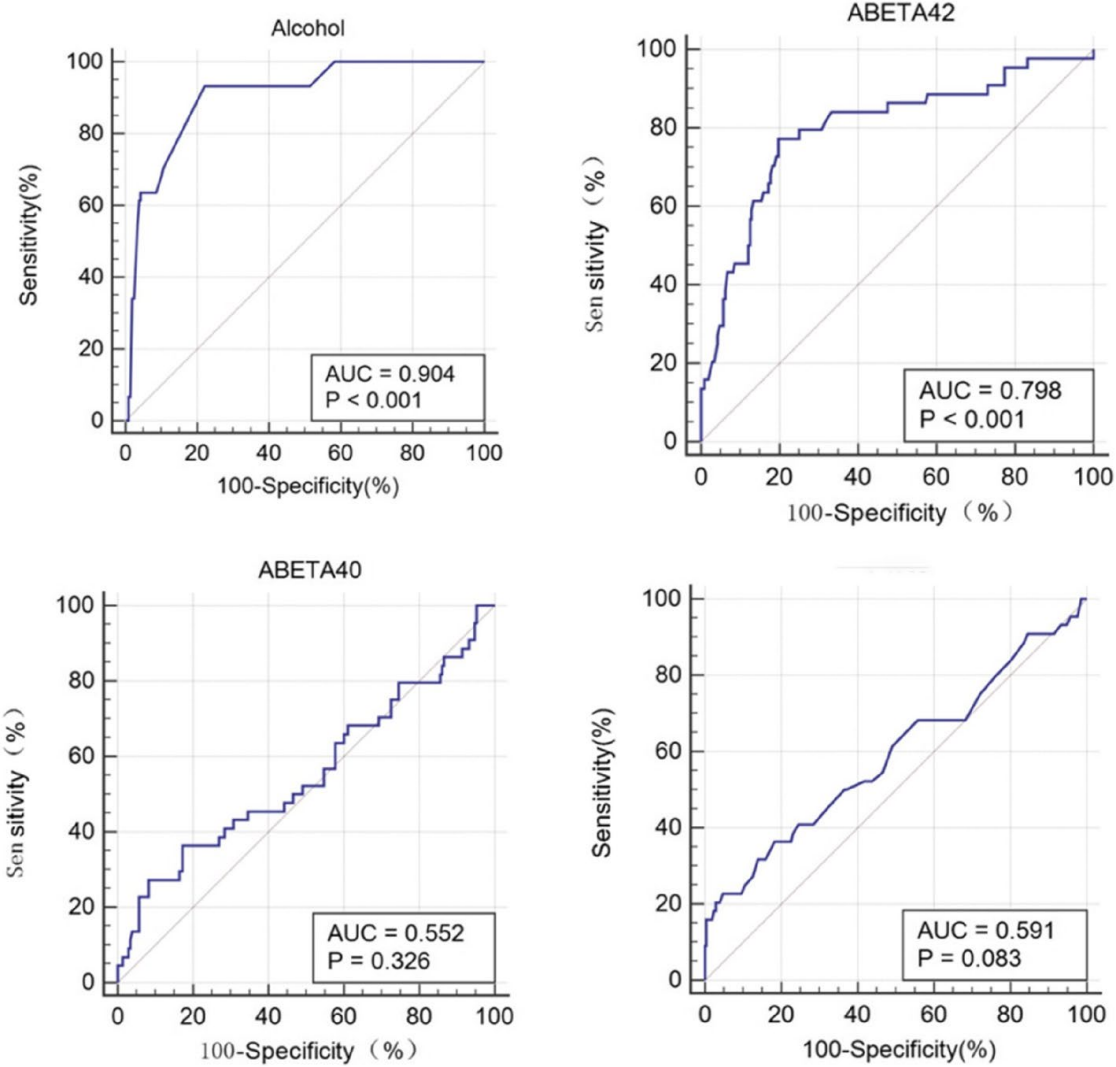
ROC curve for the probabilities from logistic modeling using alcohol, Aβ_42_, Aβ_40_, and P-tau. The results showed a high sensitivity and a low false positive rate for the use of average daily alcohol intake as a risk factor for the assessment of POD; X-axis: false positive rate, Y-axis: sensitivity

**Fig. 6:**
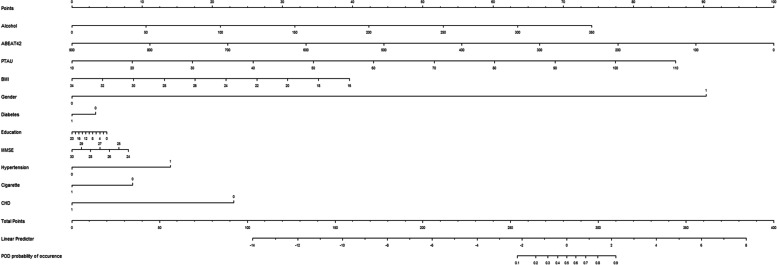
Alcohol consumption is a risk factor for POD, and within a certain range, the risk of developing POD increases as alcohol intake increases; Aβ_42_ is a protective factor for POD, and the risk of developing POD increases as the level of Aβ_42_ decreases. The process of predictors selection: Alcohol, Aβ_42_, P-tau, etc., was statistically significant with POD in this study.; Light alcohol consumption (< 12 g) in women may increase Aβ_42_ levels, so gender was also included as a predictor in the nomogram model;. BMI [44], cigarettes [45], Hypertension [46] and diabetes [47] have also been shown to be associated with the development of cognitive dysfunction

**Fig. 7:**
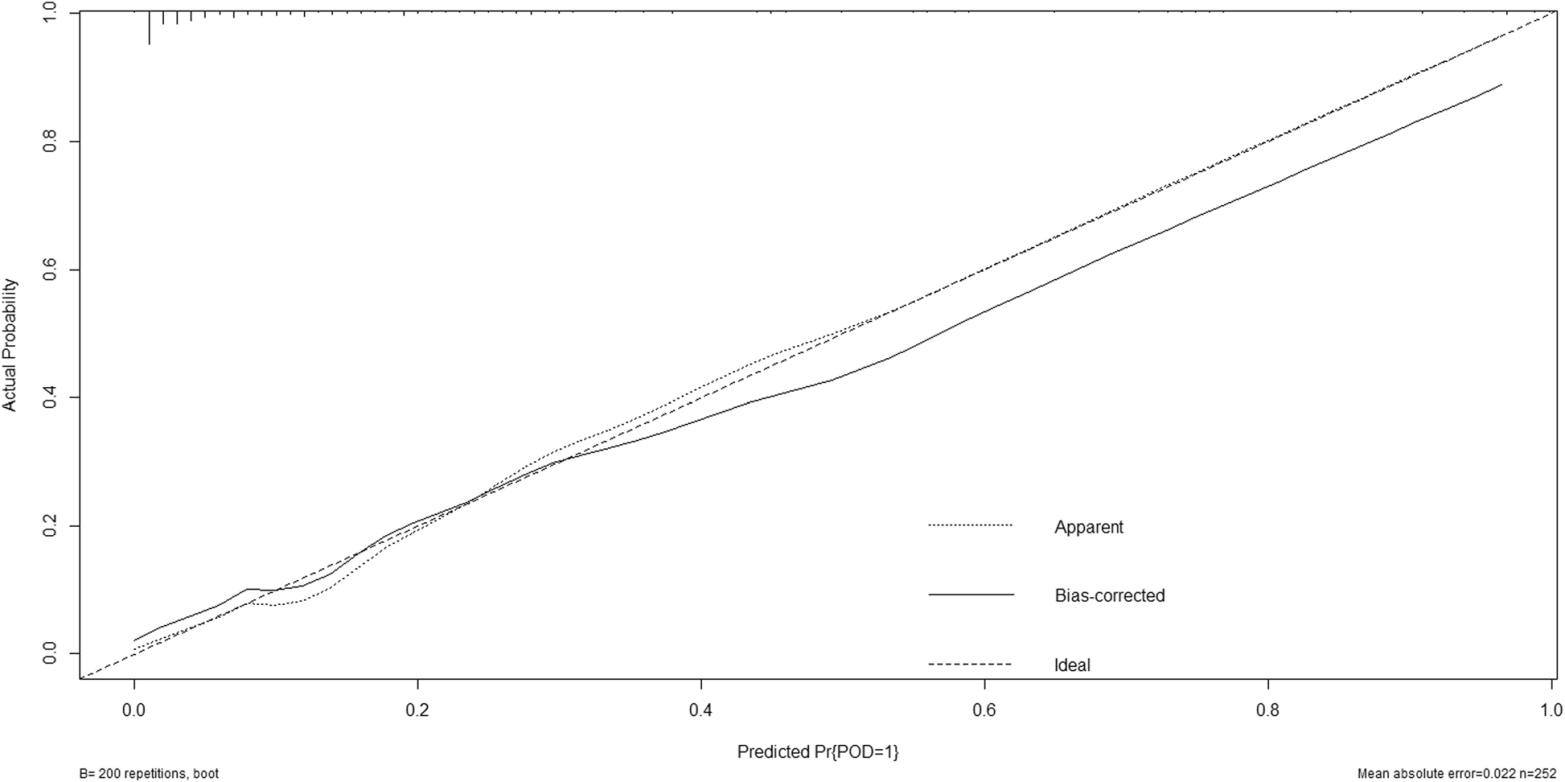
The calibration curve indicated good prediction of the nomogram (*P* = 0.797)

ROC curve showed that two factors were effective in predicting POD, alcohol consumption (AUC = 0.904; *P* < 0.001) exhibited a relatively better discriminatory ability in POD prediction compared to Aβ_42_ (AUC = 0.798; *P* < 0.001) (Fig. 5). The efficacy of each predictor is shown in the nomogram (Fig. 6). The calibration curve indicated good prediction of the nomogram (*P* = 0.797) (Fig. 7).

## Discussion

The result showed that alcohol consumption is a risk factor for POD in the elderly and possibly mediated by Aβ_42_.

POD can be defined as an acute brain dysfunction [27], Which can present as hypoactive (decreased alertness, motor activity and anhedonia), hyperactive (agitated and combative) or mixed forms [28, 29]. The presence of delirium increases the risk of developing dementia in later life [30]. Although it is usually a temporary illness with psychiatric symptoms, delirium leads to a reduced quality of life and a decreased ability to perform daily activities [31]. Moreover, POD is associated with longer hospitalization, poorer functional recovery and higher healthcare costs. 72.4% of patients will die within five years [32–34]. Thus, identifying individuals with a high risk of POD and developing early prevention and intervention strategies are significant to the public.

There are many theories about the mechanism of POD [35–37]; Aβ_40_ and Aβ_42_ are major components of senile plaques in AD. Tau is a microtubule-associated protein in neurons and is critical for microtubule formation and stability [38]. Aβ and Tau are biomarkers of plaque pathology, neurodegeneration, and neurofibrillary tangle pathology reflecting POD. Studies have demonstrated that CSF Aβ_42_, Aβ_40_, T-tau, and P-tau in patients are associated with cognitive decline [39, 40]. The study yielded similar results to previous studies, with Aβ_42_ being a protective factor for POD and P-tau being a risk factor for POD. This further validates the reliability of CSF markers as predictors of POD occurrence based on the previous.

With the development of the economy, alcohol as a globally popular beverage was consumed more often, which makes the relationship between alcohol consumption and cognitive impairment more pronounced [6, 7]. The results of this study prove that alcohol consumption is a risk factor for POD. Alcohol consumption has also been associated with cognitive decline in previous studies [41]. Cognitive decline was well documented in alcohol drinkers [18], which is consistent with the conclusion of this trial, but the underlying mechanisms need to be further discussed.

In this study, the relationship between alcohol consumption, POD and CSF markers and the possible potential mechanisms were initially explored, providing a clinical reference value for predicting and preventing POD. Epidemiological studies have shown that excessive alcohol consumption increases the accumulation of Aβ and tau protein phosphorylation [42]. What’s more, CSF indicators of amyloid accumulation were higher for those with regular alcohol consumption rather than infrequent alcohol consumption [18]. A few years ago, a study also showed a decrease in Aβ_42_ in patients with POD [12]. Interestingly, the study reached similar conclusions that alcohol consumption was an independent risk factor for POD and patients in the POD group had lower levels of Aβ_42_ than the NPOD group.

We also came up with another surprising result, alcohol intake > 24 g (heavy) is more likely to cause POD, similar to the conclusion of Richards and Sabia [41, 43]. There is a negative correlation between alcohol consumption and Aβ_42_ in POD patients, and regular alcohol drinkers have increased pathological accumulation of Aβ_42_, leading to a decreased CSF Aβ_42_ concentration [18, 42]. Sensitivity analyses yielded consistent conclusions. Combining the findings, a hypothesis that alterations in CSF Aβ_42_ might be one of the mechanisms that alcohol consumption leads to POD was made. Therefore, a mediation analysis with 10,000 bootstrapped iterations was performed to explore the mediation effects. The results suggest that in the mechanism of alcohol-induced POD generation, it may be partially mediated through Aβ_42_ (14.21%). In addition, the prediction models constructed for the high-risk factors of logistic regression analysis also indicated good sensitivity and predictability of the results of this study.

Two-way ANOVA shows a possible interaction between gender and alcohol consumption. Therefore, to exclude interactions, another simple effects analysis was performed. We found that the level of Aβ_42_ in CSF would decrease with increasing alcohol intake when “gender” was fixed. However, when the variable “daily alcohol intake” was the same, there would be no significant difference in the level of CSF Aβ_42_. Surprisingly, women had higher Aβ_42_ levels than men when the average daily alcohol intake was < 12 g (light alcohol consumption). This may suggest that light alcohol consumption in women may reduce the occurrence of POD by increasing Aβ_42_. This may be a new perspective for prevention; however, the exact mechanism needs to be verified by further studies.

The characteristic of this study is that it quantified daily drinking as alcohol intake, which increased the accuracy and reliability of the experimental results. In contrast, previous studies have only explored the current frequency of alcohol consumption but have not considered specific doses. However, this study has some limitations: firstly, it is a single-center trial, and a multicenter study is still needed. Secondly, the sample size of this study is small. Thirdly, this study focused only on the relationship between alcohol consumption and CSF biomarkers, and there may be other mechanisms. Finally, this study focused only on preoperative factors, and postoperative recovery indicators should be studied as the next step.

## Conclusion

In conclusion, alcohol consumption is a risk factor for POD in the elderly and will lead to POD through the mediation of Aβ_42_. Patients with an average daily alcohol intake of > 24 g will have an increased risk of POD. This study provides a new insight into the prevention of POD, which is expected to guide POD prevention by regulating or controlling alcohol consumption.

## Data Availability

The raw data supporting the conclusion of this article will be available by the authors, without undue reservation.

## References

[1] Rudolph JL, Marcantonio ER (2011). Review articles: postoperative delirium: acute change with long-term implications. Anesth Analg.

[2] Zenilman ME (2017). Delirium: an important postoperative complication. JAMA.

[3] Evered L, Silbert B, Knopman DS (2018). Recommendations for the nomenclature of cognitive change associated with anaesthesia and surgery-2018. Br J Anaesth.

[4] Glumac S, Kardum G, Karanovic N (2019). Postoperative cognitive decline after cardiac surgery: a narrative review of current knowledge in 2019. Med Sci Monit.

[5] Rong X, Ding ZC, Yu HD, Yao SY, Zhou ZK (2021). Risk factors of postoperative delirium in the knee and hip replacement patients: a systematic review and meta-analysis. J Orthop Surg Res.

[6] Rumgay H, Shield K, Charvat H (2021). Global burden of cancer in 2020 attributable to alcohol consumption: a population-based study. Lancet Oncol.

[7] Sousa G, Pinho C, Santos A, Abelha FJ (2017). Postoperative delirium in patients with history of alcohol abuse. Rev Esp Anestesiol Reanim.

[8] Topiwala A, Allan C, Valkanova V, et al. Moderate alcohol consumption as risk factor for adverse brain outcomes and cognitive decline: longitudinal cohort study. BMJ (Clinical research ed.). 2017;357:j2353. 10.1136/bmj.j2353 PMC5460586 28588063

[9] Wang Y, Mandelkow E (2016). Tau in physiology and pathology. Nat Rev Neurosci.

[10] Huang C, Irwin MG, Wong GTC, Chang RCC (2018). Evidence of the impact of systemic inflammation on neuroinflammation from a non-bacterial endotoxin animal model. J Neuroinflammation.

[11] Kortvelyessy P, Gukasjan A, Sweeney-Reed CM, Heinze HJ, Thurner L, Bittner DM (2015). Progranulin and amyloid-beta levels: relationship to Neuropsychology in Frontotemporal and Alzheimer’s Disease. J Alzheimers Dis.

[12] Idland AV, Wyller TB, Stoen R (2017). Preclinical amyloid-beta and Axonal Degeneration Pathology in Delirium. J Alzheimers Dis.

[13] Querfurth HW, LaFerla FM (2010). Alzheimer’s disease. N Engl J Med.

[14] Mucke L, Selkoe DJ (2012). Neurotoxicity of amyloid beta-protein: synaptic and network dysfunction. Cold Spring Harb Perspect Med.

[15] Nizynski B, Dzwolak W, Nieznanski K (2017). Amyloidogenesis of tau protein. Protein Sci.

[16] Hu X, Yang Y, Gong  DJ, NsojotINS, Neurophysiology otISoC (2017). Changes of cerebrospinal fluid Aβ, t-tau, and p-tau in Parkinson’s disease patients with cognitive impairment relative to those with normal cognition: a meta-analysis.. Neurol Sci..

[17] Xie Z, Swain CA, Ward SA (2014). Preoperative cerebrospinal fluid beta-Amyloid/Tau ratio and postoperative delirium. Ann Clin Transl Neurol.

[18] Wang Z, Li K, Tan C, et al. Associations of Alcohol Consumption with Cerebrospinal Fluid Biomarkers of Alzheimer's Disease Pathology in Cognitively Intact Older Adults: The CABLE Study. J Alzheimers Dis. 2021;82(3):1045–54. 10.3233/JAD-210140 34151793

[19] Inouye SK, van Dyck CH, Alessi CA, Balkin S, Siegal AP, Horwitz RI (1990). Clarifying confusion: the confusion assessment method. A new method for detection of delirium. Ann Intern Med.

[20] Schuurmans MJ, Deschamps PI, Markham SW, Shortridge-Baggett LM, Duursma SA (2003). The measurement of delirium: review of scales. Res Theory Nurs Pract.

[21] Shi Z, Wu Y, Li C, et al. Using the Chinese version of Memorial Delirium Assessment Scale to describe postoperative delirium after hip surgery. Front Aging Neurosci. 2014;6:297. 10.3389/fnagi.2014.00297 PMC4220661 25414664

[22] Leung J, Leung V, Leung CM, Pan PC (2008). Clinical utility and validation of two instruments (the confusion Assessment Method Algorithm and the chinese version of nursing delirium screening scale) to detect delirium in geriatric inpatients. Gen Hosp Psychiatry.

[23] Bakr A, Silva D, Cramb R, Flint G, Foroughi M (2017). Outcomes of CSF spectrophotometry in cases of suspected subarachnoid haemorrhage with negative CT: two years retrospective review in a Birmingham hospital. Br J Neurosurg.

[24] Pérez-Ruiz E, Decrop D, Ven K, et al. Digital ELISA for the quantification of attomolar concentrations of Alzheimer's disease biomarker protein Tau in biological samples. Anal Chim Acta. 2018;1015:74–81. 10.1016/j.aca.2018.02.011 29530254

[25] Pinder RM, Sandler M (2004). Alcohol, wine and mental health: focus on dementia and stroke. J Psychopharmacol.

[26] van Smeden M, Moons KG, de Groot JA (2019). Sample size for binary logistic prediction models: beyond events per variable criteria. Stat Methods Med Res.

[27] Oh ST, Park JY (2019). Postoperative delirium. Korean J Anesthesiol.

[28] Robinson TN, Raeburn CD, Tran ZV, Brenner LA, Moss M (2011). Motor subtypes of postoperative delirium in older adults. Arch Surg.

[29] Meagher D, O’Hanlon D, O’Mahony E, Casey P (2000). Trzepacz PJTJon, neurosciences c. Relatsh between symptoms motoric subtype delirium.

[30] Silverstein JH, Deiner SG (2013). Perioperative delirium and its relationship to dementia. Prog Neuropsychopharmacol Biol Psychiatry.

[31] Kijima E, Kayama T, Saito M (2020). Pre-operative hemoglobin level and use of sedative-hypnotics are independent risk factors for post-operative delirium following total knee arthroplasty. BMC Musculoskelet Disord.

[32] Marcantonio E, Flacker J, Michaels M, Resnick N (2000). Delirium is independently associated with poor functional recovery after hip fracture. J Am Geriatr Soc..

[33] Gruber-Baldini AL, Zimmerman S, Morrison RS (2003). Cognitive impairment in hip fracture patients: timing of detection and longitudinal follow-up. J Am Geriatr Soc.

[34] Lundstrom M, Edlund A, Bucht G, Karlsson S, Gustafson Y (2003). Dementia after delirium in patients with femoral neck fractures. J Am Geriatr Soc.

[35] Alam A, Hana Z, Jin Z, Suen KC, Ma D (2018). Surgery, neuroinflammation and cognitive impairment. EBioMedicine.

[36] Dutkiewicz R, Zetterberg H, Andreasson U, Blennow K, Nellgard B (2020). Dementia and CSF-biomarkers for Alzheimer’s disease predict mortality after acute hip fracture. Acta Anaesthesiol Scand.

[37] Cunningham EL, McGuinness B, McAuley DF (2019). CSF Beta-amyloid 1–42 concentration predicts Delirium following elective arthroplasty surgery in an Observational Cohort Study. Ann Surg.

[38] Guo T, Noble W, Hanger DP (2017). Roles of tau protein in health and disease. Acta Neuropathol.

[39] Ashok A, Singh N, Chaudhary S, et al. Retinal Degeneration and Alzheimer's Disease: An Evolving Link. Int J Mol Sci. 2020;21(19):7290. 10.3390/ijms21197290 PMC7582766 33023198

[40] Pradeepkiran JA, Reddy PH (2020). Defective mitophagy in Alzheimer’s disease. Ageing Res Rev.

[41] Richards M, Hardy R, Wadsworth ME (2005). Alcohol consumption and midlife cognitive change in the british 1946 birth cohort study. Alcohol Alcohol.

[42] Peng B, Yang Q, R BJ, et al. Role of Alcohol Drinking in Alzheimer's Disease, Parkinson's Disease, and Amyotrophic Lateral Sclerosis. Int J Mol Sci. 2020;21(7):2316. 10.3390/ijms21072316 PMC7177420 32230811

[43] Sabia S, Elbaz A, Britton A (2014). Alcohol consumption and cognitive decline in early old age. Neurology.

[44] Frisardi V, Solfrizzi V, Seripa D (2010). Metabolic-cognitive syndrome: a cross-talk between metabolic syndrome and Alzheimer’s disease. Ageing Res Rev.

[45] Campos MW, Serebrisky D, Castaldelli-Maia JM (2016). Smoking and cognition. Curr Drug Abuse Rev.

[46] Haslam DW, James WP, Obesity (2005). Lancet.

[47] Liu K, Song Y, Yuan Y, et al. Type 2 Diabetes Mellitus with Tight Glucose Control and Poor Pre-Injury Stair Climbing Capacity May Predict Postoperative Delirium: A Secondary Analysis. Brain Sci. 2022;12(7):951. 10.3390/brainsci12070951 PMC9317912 35884759

